# Functional robustness of adult spermatogonial stem cells after induction of hyperactive *Hras*

**DOI:** 10.1371/journal.pgen.1008139

**Published:** 2019-05-03

**Authors:** Makiko Yamada, Winson Cai, Laura A. Martin, Thierry N’Tumba-Byn, Marco Seandel

**Affiliations:** Department of Surgery, Weill Cornell Medical College, New York, New York, United States of America; Cornell University, UNITED STATES

## Abstract

Accumulating evidence indicates that paternal age correlates with disease risk in children. De novo gain-of-function mutations in the FGF-RAS-MAPK signaling pathway are known to cause a subset of genetic diseases associated with advanced paternal age, such as Apert syndrome, achondroplasia, Noonan syndrome, and Costello syndrome. It has been hypothesized that adult spermatogonial stem cells with pathogenic mutations are clonally expanded over time and propagate the mutations to offspring. However, no model system exists to interrogate mammalian germline stem cell competition in vivo. In this study, we created a lineage tracing system, which enabled undifferentiated spermatogonia with endogenous expression of *Hras*^*G12V*^, a known pathogenic gain-of-function mutation in RAS-MAPK signaling, to compete with their wild-type counterparts in the mouse testis. Over a year of fate analysis, neither *Hras*^*G12V*^-positive germ cells nor sperm exhibited a significant expansion compared to wild-type neighbors. Short-term stem cell capacity as measured by transplantation analysis was also comparable between wild-type and mutant groups. Furthermore, although constitutively active HRAS was detectable in the mutant cell lines, they did not exhibit a proliferative advantage or an enhanced response to agonist-evoked pERK signaling. These in vivo and in vitro results suggest that mouse spermatogonial stem cells are functionally resistant to a heterozygous *Hras*^*G12V*^ mutation in the endogenous locus and that mechanisms could exist to prevent such harmful mutations from being expanded and transmitted to the next generation.

## Author summary

Recent research has found that advanced paternal age is associated with increased risk in children to develop a subset of congenital anomalies, such as Apert syndrome, achondroplasia, Noonan syndrome, and Costello syndrome. The causative genetic errors (mutations) in these disorders have been identified to originate from the fathers’ testicles and their numbers increase with fathers’ age. It has been hypothesized that the germline stem cells that continuously self-renew and differentiate to supply sperm (referred as spermatogonial stem cells [SSCs]) carry these mutations and have the ability to expand preferentially as compared to normal SSCs with advancing age of the father, thereby increasing the likelihood of transmission of mutant sperm to the next generation. To test this hypothesis, we created a mouse model, in which a mutation known to enhance cell proliferation is induced in a subset of SSCs, and these cells compete with the neighboring normal (i.e., wild-type) stem cells. However, surprisingly, the germline cell population carrying the mutation in the testis was stable over a year of observation, suggesting that mechanisms could exist to prevent such harmful mutations from being expanded and transmitted to the next generation.

## Introduction

In order to propagate genetic information to the next generation with high fidelity, germline cells must maintain a low mutation rate. Nevertheless, maternal germline cells (human oocytes) are well known to transmit abnormal chromosomes to offspring, especially in advanced maternal age (reviewed in [1]). Surprisingly, recent high-throughput genome analyses have revealed that men contribute a much higher number of mutations, specifically de novo single nucleotide mutations, to their children than do women [2–4]. Most strikingly, the risk of certain genetic disorders increases with advancing age of the father at the time conception of the child, referred to as the paternal age effect (PAE). This phenomenon could be explained by the unique biology of paternal germline stem cells. The latter are termed spermatogonial stem cells (SSCs), and, once established in the post-natal period, continue to self-renew and differentiate to supply sperm in mammals throughout adult life. This continuous self-renewal and long-term survival of SSCs may underlie the increase in mutation burden with paternal age, due to a cumulative increase in copy errors or other DNA lesions, despite the fact that the baseline germline mutation rate is thought to be lower than that of somatic cells [5]. Although the natural history of mutations in the aging testis is poorly understood, pathogenic variants are occasionally transmitted to offspring, resulting in a wide range of disorders. Among these, de novo gain-of-function mutations in the growth factor receptor-RAS signaling pathway are classically known to cause so-called PAE disorders, such as Apert syndrome, achondroplasia, Noonan syndrome, and Costello syndrome (reviewed in [6]).

Direct quantification of such mutations in the sperm and testes of healthy men of different ages has revealed an age-dependent increase in the mutation burden, in a manner that exceeds what would be expected from cumulative copy errors [7–9]. Moreover, in human testes, Ras pathway-associated mutations have been reported to occur in a clustered manner, suggesting that SSCs with PAE mutations are positively selected and clonally expand in normal, otherwise healthy testes over time [10–12]. We previously showed that a gain-of-function mutation in FGFR2 that causes Apert syndrome is sufficient to confer a selective advantage to murine SSCs in vitro [13]. However, no model system has been developed to interrogate mammalian SSC competition in vivo. Furthermore, no cell biological or molecular mechanisms have been described to explain this phenomenon. Although clonal expansion of stem cells with oncogenic mutations has been observed in the mouse intestinal crypt model [14, 15], it is not clear whether the same holds true for SSCs in the adult mouse testis. To test this long-standing hypothesis for SSC competition, we sought to establish an inducible mosaic model in which a hyperactive form of *Hras* could be induced within the endogenous locus in a subset of SSCs so that their long-term fate could be followed.

The undifferentiated spermatogonia (A_undiff_) represent a population of cells in the mammalian testes that is defined by morphology and function. Along with somewhat more committed cells, the A_undiff_ pool contains long-term self-renewing SSCs. Morphologically, the A_undiff_ in rodents comprises A_s_ (single), A_pr_ (pair), and A_al_ (aligned) cells, which are remarkably interconvertible, with significant migratory capacity and cell fate plasticity when subject to stress [16, 17]. Those cells reside along the basement membrane in the seminiferous tubules and are heterogeneous with respect to expression of genetic markers. Hara et al. (2014) first employed a cre driver controlled by the endogenous promoter of *Gfra1*, one of the robustly expressed markers for A_undiff_ and demonstrated that the labeled population marked by *Gfra1-creER*^*T2*^ comprised the long-term stem cell fraction [16]. Therefore, in our current study, we chose the same *Gfra1* cre driver to create a novel germline mosaic model.

HRAS, a member of the RAS oncogene superfamily, is a monomeric GTPase and relays signals from receptor tyrosine kinases to the cell interior. It serves as a molecular switch for a MAP kinase signaling module in which HRAS is “on” when GTP is bound and “off” when GDP is bound (as reviewed in [18]). The *Hras*^*G12V*^ mutation encodes a hyperactive form of HRAS protein that is locked in a GTP-bound state and cannot hydrolyze its bound GTP to GDP [19, 20]. *Hras*^*G12V*^ is a rare mutation found in patients with Costello syndrome, whereas the *Hras*^*G12S*^ mutation comprises the majority of probands [21], and it has been demonstrated that the *Hras* mutation burden in sperm from healthy donors increases according to donors’ age [9]. In mice, heterozygous *Hras*^*G12V*^ mice phenocopy human Costello syndrome [22]. It was observed that overexpression of *Hras*^*G12V*^ using transgenes in cultured mouse SSCs caused tumor development [23]. However, the effect of one copy of hyperactive Hras expression on paternal SSCs in vivo, simulating the putative earliest events in the human gain-of-function mutation disorder, has never been addressed.

In order to understand how a hyperactive HRAS affects long-term paternal stem cell fate, we induced *Hras*^*G12V*^ at the endogenous locus in A_undiff_ in a mosaic manner. We found that *Gfra1-creER*^*T2*^ successfully drives mosaic *Hras*^*G12V*^ activation in the adult germline in vivo. This model system allowed us to track mutated cell fate for prolonged chase periods in a quantitative manner. Surprisingly, the mutated SSC fraction persisted stably without significant expansion, suggesting that robust mechanisms exist to protect the SSC pool from harmful expansion of mutated cells and prevent transmission of deleterious alleles.

## Results

### Mosaic induction of endogenous *Hras*^*G12V*^ alleles in the undifferentiated spermatogonial pool

**Fig 1:**
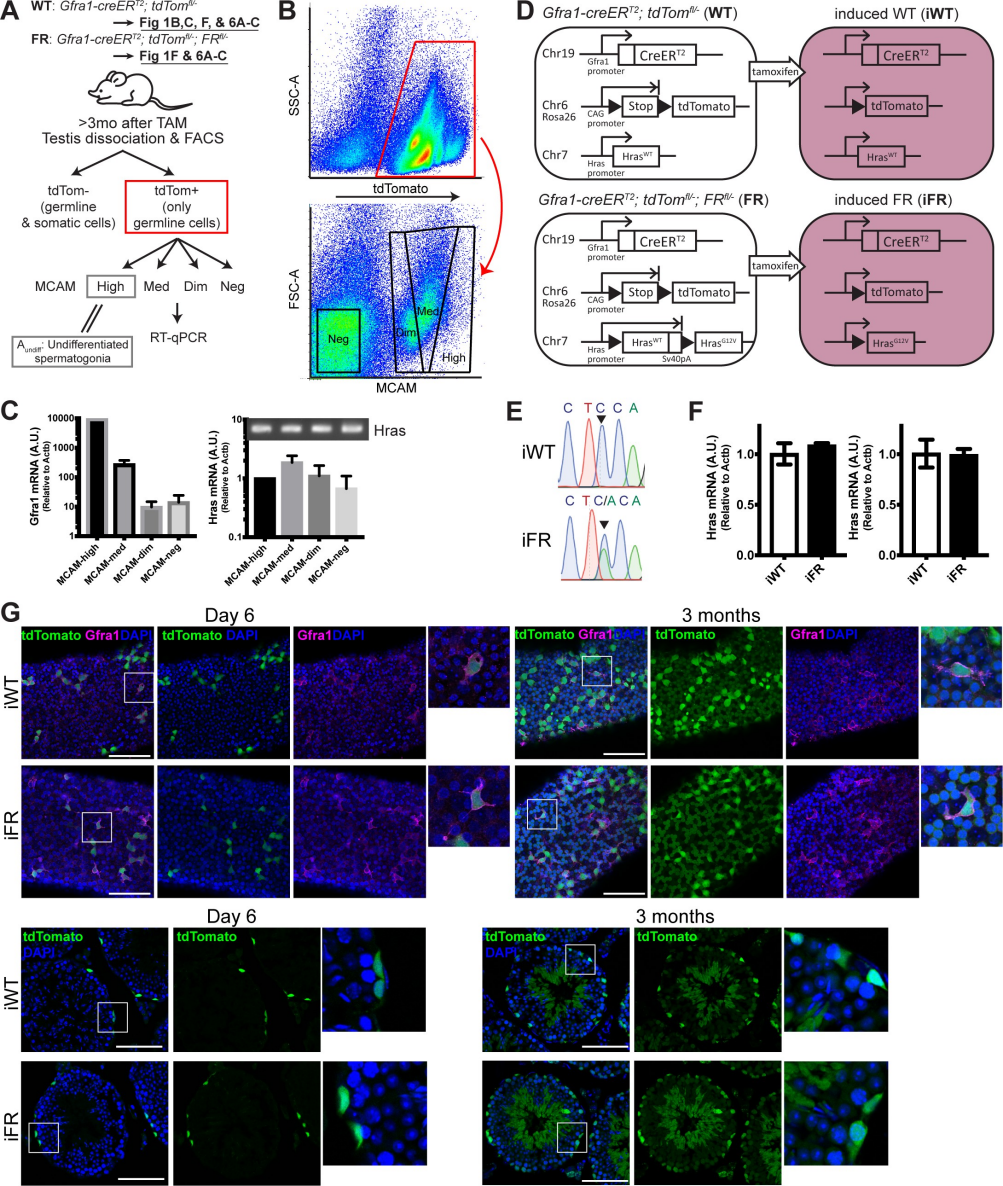
Establishment of an inducible mosaic model. A. Schematic showing workflow for collection of the A_undiff_ population from dissociated testicular cells of genetically engineered mice with induced germ cell-restricted tdTomato expression with or without the *Hras*^*G12V*^ allele. After 3–4 months of tamoxifen administration to *Gfra1-creER*^*T2*^; *tdTom*^*fl/-*^ (WT) mice, testes were dissociated, and the tdTomato+ cells were sorted based on MCAM expression levels (high, medium, dim, and negative). B. Flow cytometry analysis of dissociated testicular cells from induced WT mice. After exclusion of doublet and dead (DAPI+) cells, tdTomato+ gated cells (top) were sorted to obtain MCAM high, medium, dim, and negative populations (bottom). C. *Gfra1* and *Hras* transcripts in different germ line populations of the wild-type testis. Using tamoxifen-induced *Gfra1-creER*^*T2*^; *tdTom*^*fl/-*^ animals (n = 3), the tdTomato+ population which contains only germline cells) was sorted into MCAM-high, medium, dim, and negative populations. The MCAM-high population yielded elevated *Gfra1* mRNA (left graph), indicating that this population is enriched for undifferentiated spermatogonia (A_undiff_). The level of *Hras* transcript (relative to *Actb*) was similar among these four different germline populations. Inset: the final qPCR products were run on agarose gel and shown on the top. D. Strategy to induce *Hras*^G12V^ exclusively in undifferentiated spermatogonia. After tamoxifen administration, the wild-type *Hras* allele is excised as it is flanked by two loxP sites (black triangle). Both the wild-type *Hras* allele (before recombination) and the recombined *Hras*^*G12V*^ alleles are under the regulatory control of the endogenous *Hras* promoter. After tamoxifen administration, we designated the animals as induced WT (iWT) or induced FR (iFR). E. Sanger sequence trace of reverse-transcribed PCR product from tdTomato+ germ line cells in testis of iWT and iFR three months after a four-day tamoxifen regimen. Note that the mutant A peak is smaller than the C peak, indicating that not all the tdTomato+ cells were positive for *Hras*^*G12V*^. F. Similar *Hras* mRNA levels in A_undiff_ from iWT and iFR (n = two littermates; right and left). RT-qPCR primers were designed to recognize a common region for iWT & iFR. Animals were treated with two courses of tamoxifen (one course = 4 consecutive days) over two weeks. G. Reporter expression in the undifferentiated spermatogonia after one course of tamoxifen. Top panels are whole-mount immunofluorescence using anti-GFRA1 (pseudocolored magenta). On day 6 (day 0 = first day of tamoxifen administration), tdTomato (pseudocolored green) efficiently labeled undifferentiated spermatogonia positive for GFRA1 (magenta) both in iWT and iFR (representative images from n = 2 animals for each group). A representative A _single_ cell positive for GFRA1 is magnified from the white box. After 3 months, whole segments were occupied by tdTomato+ germline cells. Bottom panels are cross-sectioned tubules showing that tdTomato+ A_undiff_ differentiate and produce spermatids both in iWT and iFR. Scale bars: 100 μm.

Whereas cultured SSCs are reported to express *Hras* [23], the abundance of *Hras* transcripts in the A_undiff_ spermatogonia *in vivo* has not been reported. Therefore, we evaluated *Hras* expression *in vivo* in A_undiff_, as a surrogate for SSCs. To isolate the A_undiff_ population by flow sorting, we stained dissociated testicular cells with an anti-MCAM antibody (Fig 1A and 1B). Although MCAM-based sorting has been previously shown to enrich the A_undiff_ population, MCAM is also expressed in somatic cells [24, 25]. To avoid somatic cell contamination in FACS experiments, we first obtained tdTomato-labeled germ line cells using *Gfra1-creER*^*T2*^; *tdTom*^*fl/-*^ mice, in which tamoxifen induces tdTomato efficiently in the A_undiff_ and their progeny but not in testicular somatic cells (Fig 1G, iWT). Three months after tamoxifen induction, tdTomato+ cells that had high, medium, low, or absent (negative) expression of MCAM were isolated by FACS and analyzed for *Hras* by RT-PCR (Fig 1A and 1B). *Hras* transcripts were found throughout the different germ cell populations tested (Fig 1C).

To induce the *Hras*^*G12V*^ mutation exclusively in A_undiff_, we employed the *Gfra1-creER*^*T2*^ mouse line. A “flox-and-replace” (FR) model generated by Chen et al. (2006) was utilized to produce monoallelic *Hras*^*G12V*^ (Fig 1D) [22]. In this mouse line, the endogenous *Hras* locus consists of 2 tandemly-arrayed *Hras* genes, the upstream of which is a WT allele flanked by *loxP* sites, such that the downstream *Hras*^*G12V*^ allele is silent until removal of the upstream 2.5 kb WT gene by recombination. By breeding, we generated *Gfra1-creER*^*T2*^; *tdTom*^*fl/-*^; *FR-Hras*^*G12Vfl/-*^ mice. Tamoxifen-inducible Cre-mediated recombination of the *Hras* locus resulted in conversion from wild-type *Hras* to *Hras*^*G12V*^ and activation of tdTomato expression at the *Rosa26* locus. (Fig 1D & 1G). To verify the presence of the mutation in the testes, cDNA was obtained from FACS-sorted tdTomato-positive testicular cells, which consisted of pure germline cells (since the *Gfra1* promoter is not active in testicular somatic cells) and was amplified for sequencing. Sanger sequencing confirmed that the expected nucleotide substitution (C>A) was present (Fig 1E). From the chromatogram, not all the tdTomato-positive cells exhibited *Hras*^*G12V*^ three months after tamoxifen induction, indicating that wild-type and *Hras*^G12V^-positive germline cells coexisted in a mosaic manner in the labeled germline cell population. Importantly, *Hras* mRNA levels of A_undiff_ in vivo (i.e., tdTomato+/MCAM-high population in Fig 1A) measured by qPCR were similar between induced WT and FR mice, validating that a physiological level of *Hras* expression (WT + *G12V*) was achieved in the induced FR (iFR) model by utilizing the endogenous locus (Fig 1F).

**Fig 2:**
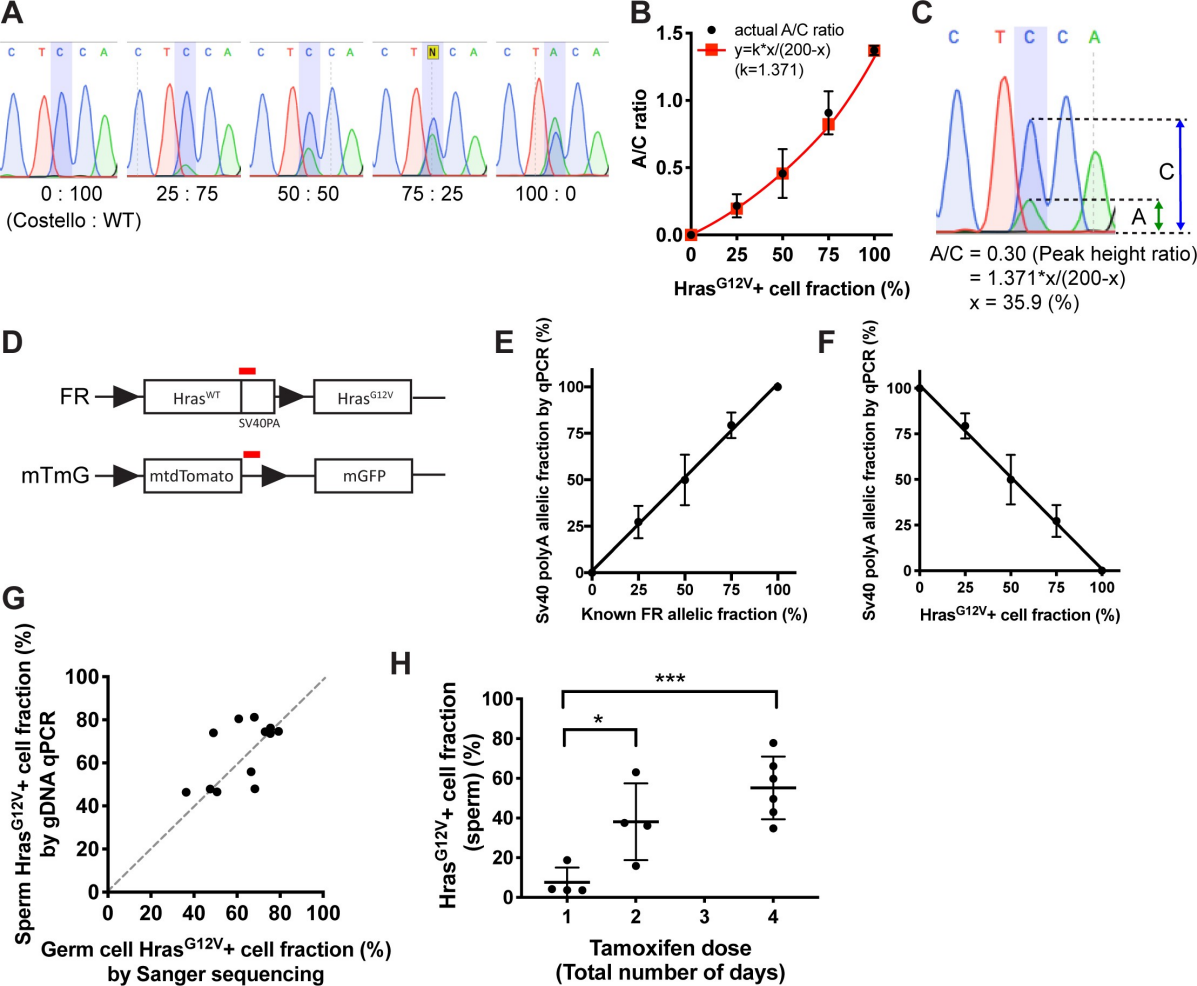
Quantification of *Hras*^*G12V*^ fractional abundance. A. Five representative chromatograms from Sanger sequencing. Costello and WT offspring RNA were mixed at varying ratios (0–100%), reverse-transcribed into cDNA, amplified by PCR, and sequenced. N≥3 mouse pairs were used for each concentration. B. Generation of a model curve for quantification of variant nucleotide. To quantify the mutated nucleotide (cytosine→adenine) from Sanger sequencing traces, a model curve (See S1 Fig) was proposed as y = k*x/(200-x) in which *x* is *Hras*G12+ cell fraction, *y* is A/C ratio, and *k* is a constant for PCR efficiency difference between WT and *Hras*^*G12V*^ templates. By using the actual A/C ratios obtained by Fig 1A, we confirmed *k* = 1.371. C. An example of calculation for the *Hras*^G12V^+ cell fraction: *x*%. Peak heights of A and C were measured by image analysis (ImageJ) and A/C = 0.30 was obtained. Using the equation: *y = 1*.371*x/(200-x), *x* = 35.9 (%) was obtained. D. Quantification of *Hras*^G12V^+ cell fraction using gDNA. gDNA qPCR primers were designed to amplify the red regions of SV40 polyA and the end of *mtdTomato* alleles respectively. Tamoxifen-inducible Cre-mediated recombination results in deletion of these regions. E. By running qPCR with mixed sperm gDNA of FR heterozygote (without recombination) and wild-type sperm at varying ratios (x-axis: 0, 25, 50, 75, 100%: n = 3 samples per each), we confirmed the linear relationship between the known FR allele+ cell fraction (*x*%) and SV40 polyA allelic fraction (*y*%) obtained by qPCR (*y = x*). F. Loss of SV40 PA implies a gain of *Hras*^*G12V*^, indicating that the SV40 PA allelic fraction (y%) is in inverse proportion to the *Hras*^*G12V*^-positive cell fraction (*z*), (*y = 100-z*). G. Scatter plot showing the correlation between the germ cell *Hras*^G12V^-positive cell fraction by Sanger sequencing and the sperm *Hras*^G12V^-positive cell fraction by gDNA qPCR. Each dot represents one animal (n = 12). A significant correlation was observed (R^2^ = 0.349, P [two-tailed] = 0.0437). H. Tamoxifen dose-dependent increase in *Hras*^G12V^-positive cell fraction. After 3 months from tamoxifen administration, sperm from the cauda epididymis was sampled, and the *Hras*^G12V^-positive cell fraction was calculated by gDNA qPCR. Tamoxifen (100mg/kg) was administered either one, two, or 4 days, consecutively (n≥4 mice per dose). The *Hras*^G12V^-positive cell fraction increases as the dose (number of days) increases. T-test was performed to compare the two doses (*P<0.05, ***P<0.001).

To measure the fractional abundance of *Hras*^G12V^-positive cells in germline cells, two independent methods were developed (Fig 2A–2F). First, Sanger sequencing-derived plots of amplified cDNA from RNA were employed to quantify the mutated nucleotide (cytosine→adenine) from sequencing traces and create a model equation (S1 Fig). The validity of the model was confirmed by amplifying cDNA samples of RNA of known standard concentrations from WT (*Hras*^WT/WT^ or *Hras*^FR/WT^) and Costello syndrome (*Hras*^*G12V/WT*^) mice at varying ratios (0–100% Costello mRNA) (Fig 2A–2C). The relationship between the *Hras*^G12V^-positive cell fraction (*x*%) and the ratio of adenine/cytosine (A/C) peak heights (*y*) fitted the model curve (*y = 1*.371*x / [200-x]). In the second approach, sperm genomic DNA (gDNA) analysis was used to quantify the *Hras*^G12V^-positive cell fraction. GDNA qPCR primers were designed to detect an *SV40* poly-A (PA) region exclusive to the *FR-Hras*^*G12Vfl*^ locus but which is lost after recombination (Fig 2D–2F). By performing qPCR with mixed gDNA comprising heterozygotic *FR-Hras*^*G12V*^ (i.e., without recombination) and wild-type sperm at different known ratios, we confirmed a linear relationship between the *FR-Hras*^*G12Vfl*^ recombined fraction (*x*%) and the *SV40* PA allelic fraction obtained by qPCR (*y*%; Fig 2E). Since the loss of *SV40* PA or *FR* means a gain of *Hras*^*G12*V^, *y = 100-z* was obtained, where *z* is the *Hras*^G12V^-positive cell fraction (Fig 2F). Using these two methods, *Hras*^G12V^-positive cell fractions of tamoxifen-induced animals were measured. The calculated fractions from sperm gDNA qPCR correlated well with that from the Sanger sequencing (Fig 2G). Furthermore, we observed a tamoxifen dose-dependent increase in the *Hras*^G12V^-positive fraction in sperm gDNA (Fig 2H). These results indicate not only that *Hras*^G12V^ was successfully induced in A_undiff_ by tamoxifen but also that *Hras*^G12V^ A_undiff_ can undergo differentiation and produce sperm.

**Fig 3:**
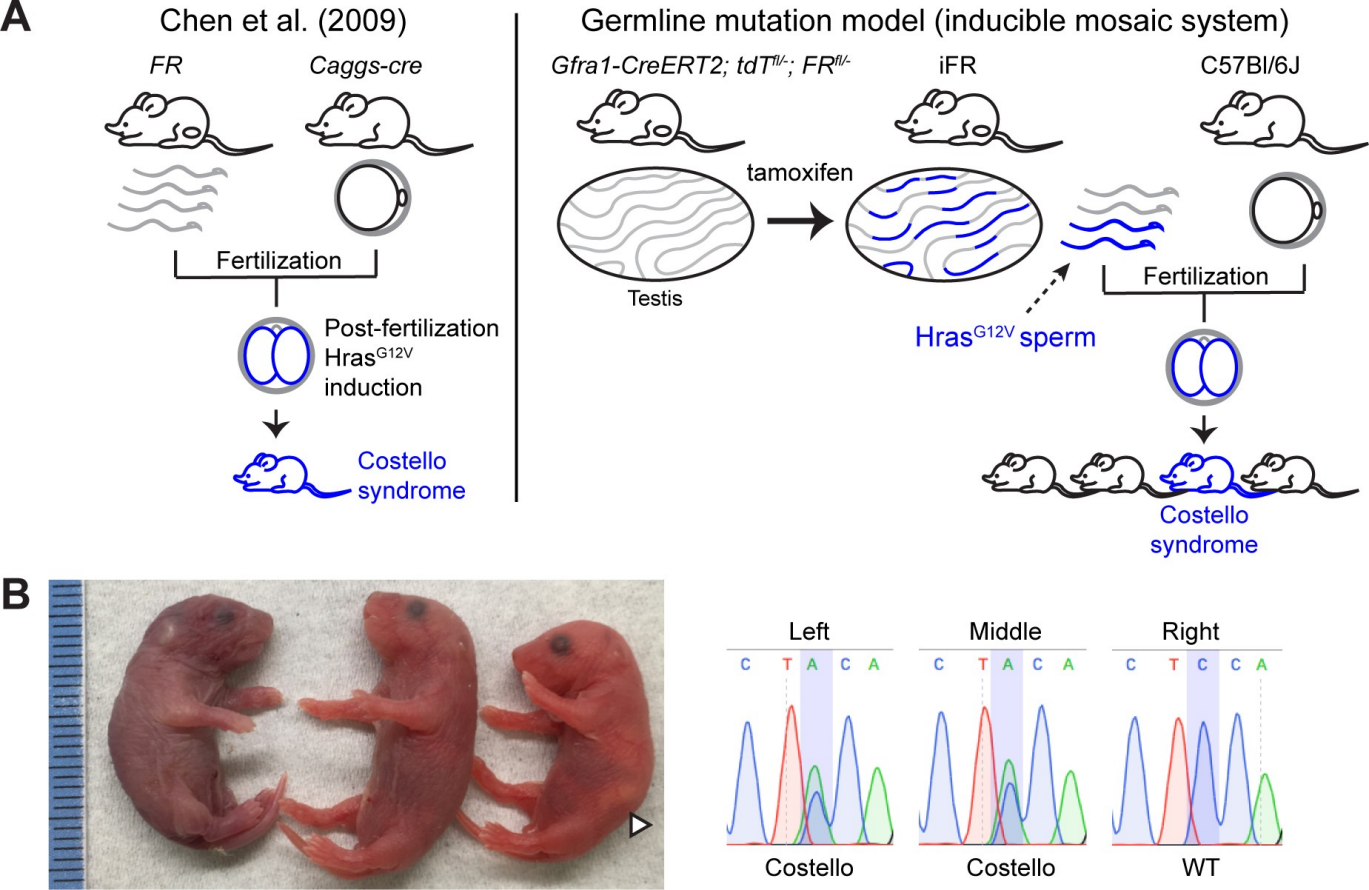
Generation of offspring with Costello syndrome. A. Germline mutation models. The previous model (left) generated by Chen et al. (2009) induces *Hras*^*G12V*^ after fertilization. The mosaic germline mutation model (right) induces mutations in a fraction of the undifferentiated spermatogonia and enables functional studies of mutant cells among wild-type neighbors and the propagation of the mutation induced in the stem cells to subsequent generations. B. Postnatal day 0 pups born from iFR (male) x C57 (female). Genotyping using RNA revealed that the left two were Costello (*Hras*^*G12V*^) offspring and the right was wild-type, whose corresponding Sanger sequencing results are shown on the right. The left pup was found dead. The middle one was cyanotic and did not have a milk spot, whereas the right wild-type was pink and showing a milk spot (white arrow head).

Previously, Chen et al. (2009) used their murine *FR-Hras*^*G12V*^ allele to model human Costello syndrome offspring [22] (Fig 3A left). In that study, *FR-Hras*^*G12V*^ mice were crossed with *Caggs-Cre* (*CC*) mice to obtain *Hras*^*G12V*^ heterozygotes, which phenotypically recapitulated Costello syndrome (driven by *Hras*^*G12S*^). In the prior model, *Hras*^G12V^ induction takes place after fertilization, rather than in the parental germ cells. On the other hand, our inducible system enabled us to test whether mosaic *Hras*^G12V^-positive A_undiff_ (in a context of neighboring wild-type germ cells and somatic cells) can differentiate into normal sperm and give rise to F1 offspring with Costello syndrome (Fig 3A right). Tamoxifen-induced *Gfra1-creER*^*T2*^; *tdTom*^*fl/-*^; *FR*^*fl/-*^ males (n = 3) were crossed with C57Bl/6J females and their pups were sacrificed at post-natal day 0 and genotyped using RNA. Genotyping revealed that Costello offspring were indeed born (left two pups in Fig 3B). This result demonstrates that *Hras*^G12V^-positive A_undiff_ are able to self-renew, undergo differentiation, and give rise to offspring.

### Stability of the *Hras*^*G12V*^ mutation burden over time

**Fig 4:**
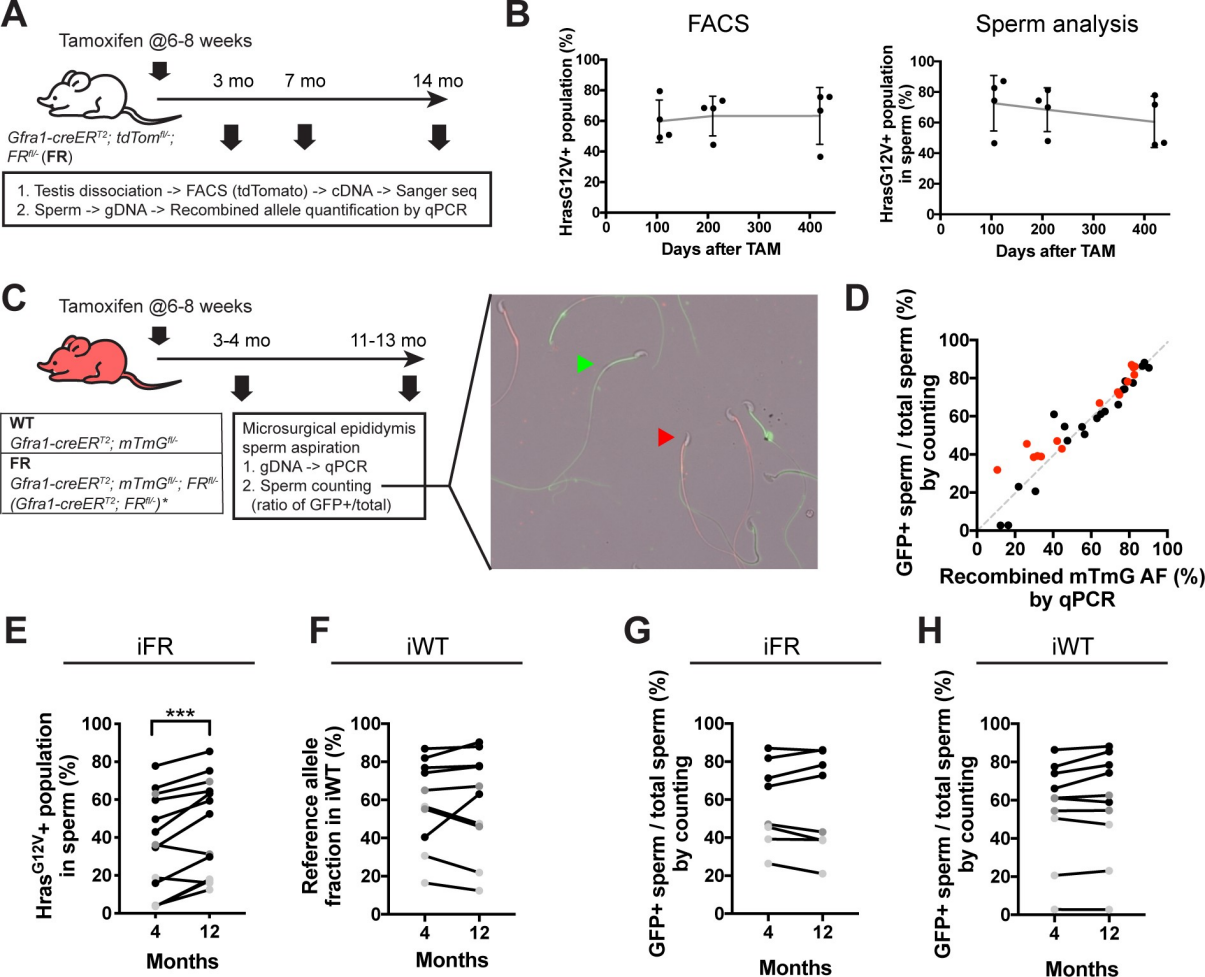
Temporal change in the *Hras*^G12V^-positive cell fraction. A. Experimental strategy for lineage tracing. At 6–8 weeks of age, FR mice (n = 4 mice at each timepoint) were treated with tamoxifen (100 mg/kg) for 4 days and sacrificed to obtain tdTomato+ germline cells by FACS and sperm analysis at 3, 7, and 14 months after tamoxifen. The two independent methods, Sanger sequencing and gDNA qPCR (described in Fig 2A–2G), were employed to calculate the *Hras*^G12V^-positive cell fraction. B. (left) Proportion of *Hras*^G12V^-positive cells in the labeled (tdTomato+) germline population over time (obtained by FACS). B. (right) *Hras*^G12V^-positive sperm proportion. C. Experimental strategy for serial sperm collection and a representative image of collected sperm (right). Microsurgical epididymis sperm aspiration was repeated twice in the same individuals at 3–4 months and 11–13 months. At each time point, gDNA qPCR was performed to measure the *Hras*^*G12V*^+ cell fraction and the reference allelic fraction. For qPCR of *SV40 PA*, *Gfra1-creER*^*T2*^; *FR*^*fl/-*^ mice were also used (*). Additionally, GFP+ and tdTomato+ spermatids were counted to calculate the ratio of GFP+ sperm to total sperm (GFP+ plus tdTom+). The green and red arrowheads indicate representative GFP+ and tdTomato+ spermatids, respectively. D. Scatter plot showing the correlation between the cell fraction of the recombined *mTmG* and GFP+ sperm ratio. One dot corresponds to one sample. A strong correlation was found for the qPCR-measured AF of recombined *mTmG* and the ratio of manually counted GFP+ sperm to total sperm (r^2^ = 0.9008, p<0.0001). Black = WT. Red = MUT. E. Fraction of *Hras*^G12V^-positive population in sperm at 4 and 12 months after tamoxifen (paired t-test, ***p<0.001, n = 13 pairs). Sperm was collected twice within the same animals, and the gDNA was extracted for qPCR. Black dots represent animals treated with tamoxifen for 4 days; gray dots for 2 days; and light gray dots for 1 day (same for F, G, and H). F. Fraction of *mTmG*-recombined allele in iWT. No change was observed in the fraction over time (paired t-test, P = 0.82, n = 10 pairs). G. Fraction of GFP+ sperm out of total sperm from iFR. No change was observed from 4 to 12 months (paired t-test, P = 0.94, n = 8 pairs). H. Fraction of GFP+ sperm out of total sperm from iWT. No change was observed from 4 to 12 months (paired t-test, P = 0.12, n = 10 pairs).

*Hras*^*G12V*^ has been detected in human sperm of healthy donors and is allelic to *Hras*^*G12S*^, the most common mutation in Costello syndrome, which increases with sperm donor age [9]. Given that gain-of-function mutations in the RAS pathway mediate positive selection of human SSCs [26, 27] and that overexpression of an *Hras*^*G12V*^ cDNA is tumorigenic [23], we hypothesized that an increasing burden of *Hras*^*G12V*^ would be detectable over time following induction in the adult testis. To test this hypothesis, our inducible mutation model enabled us to follow the fate of *Hras*^G12V^-positive A_undiff_ over time. The proportion of *Hras*^G12V^-positive cells in the labeled germline population was assessed at different time points after tamoxifen administration (Fig 4A). Sanger sequencing showed that the *Hras*^*G12V*^ allele fraction did not increase from 3 to 14 months (Fig 4B, left). Notably, despite the long chase period, no germ cell tumors were detected in these animals, and the testes were normal at the gross histological level (see Fig 1G).

Sperm mutation analysis serves as a more physiologically relevant readout, since human studies on mutation burden utilized sperm samples from different age individuals [7, 9]. Thus, to mirror the human studies, we collected sperm from different time points following tamoxifen induction. Using qPCR, we quantified the *Hras*^G12V^-positive cell fractions in gDNA of sperm obtained from the cauda epididymis at these time points. Similar to the *Hras*^G12V^-positive cell frequency among tdTomato-labeled germ cells, the *Hras*^G12V^-positive sperm proportion also did not show an increase over the 11-month chase period (Fig 4B, right).

Sperm studies have demonstrated large inter-individual variance in the mutation burden in the same age cohort [7, 9]. Therefore, such static observations require a large sample size, which is not always readily achieved. Furthermore, the time at which mutations first appear in the human testis is unknown, nor is the mutational load at relevant loci at the time of early adulthood. An ideal study would examine sperm serially from same individuals, in order to capture a temporal change in the proportion of the variant cell population using a relatively small cohort. If selection for the *Hras*^*G12V*^ genotype were to occur, one would predict an increase in the same individual over time. Thus, we established a method for in vivo serial sperm sampling. We performed microsurgical epididymal sperm aspiration [28], in which sperm was withdrawn twice from a live single male from the ipsilateral testis (Fig 4C & S2 Fig). For this study, we employed the *mTmG* reporter mouse line, in which membrane tdTomato expression converts to membrane GFP (mGFP) upon cre-induced recombination [29]. We generated *Gfra1-creER*^*T2*^; *mTmG*^*fl/-*^; *FR-Hras*^*G12Vfl/-*^ males and induced *Hras*^G12V^ and mGFP in A_undiff_ (Fig 4C). Sperm from wild-type siblings (i.e., *Gfra1-creER*^*T2*^; *mTmG*^*fl/-*^) served as controls. In the control model, the recombined *mTmG* fraction obtained by measuring the loss of the *tdTomato* allele (as a reference) by qPCR should not change over time unless there is competition between tdTomato+ and GFP+ A_undiff_. Both the *Hras*^*G12V*^ and reference allelic fractions were measured by qPCR as above (see Fig 2D). From 4 months to 12 months, a minimal increase (~9% on average) in the *Hras*^G12V^-positive cell fraction was observed (Fig 4E). Of note, the reference alleles in the wild type did not show any increase over the same time period, suggesting that tdTomato+ and GFP+ germ cells are mutually neutral with respect to fitness (Fig 4F).

To confirm whether the *Hras*^*G12V*^-positive cell fraction increases, we used a different method to assess the ratios of sperm bearing mGFP vs. tdTomato, resulting from *Gfra1*-expressing progenitors, in which the reporter transgene either remained intact or, alternatively, underwent recombination. The mGFP+ and tdTomato+ sperm were counted at two time points, at 4 months and 12 months, and the ratio of mGFP-positive to total sperm (mGFP-positive plus tdTomato-positive) was calculated. There was a strong positive correlation between values obtained by sperm counting vs. qPCR with the r^2^ = 0.90 (p<0.0001), validating that the sperm counting method itself was as accurate as the qPCR (Fig 4D). Thus, if the *Hras*^*G12V*^-positive sperm increase over time, we should observe an increase in the GFP+ sperm population. However, no change in the fraction of mGFP+ sperm in the mutant was detected (Fig 4G). Finally, we analyzed successive litters from tamoxifen-induced *Gfra1-creER*^*T2*^; *tdTom*^*fl/-*^; *FR-Hras*^*G12Vfl/-*^ sires; no increase was observed in the proportion of *Hras*^*G12V/WT*^ offspring obtained at the early phase of the study interval compared to the late phase (S1 Table). Taken together, these data suggest that the population structure is relatively stable and do not support a substantial change in the ratios of controls and mutant cells within the mosaic pool.

### Comparable stem cell activity between wild-type and *Hras*^*G12V*^-positive undifferentiated spermatogonia

**Fig 5:**
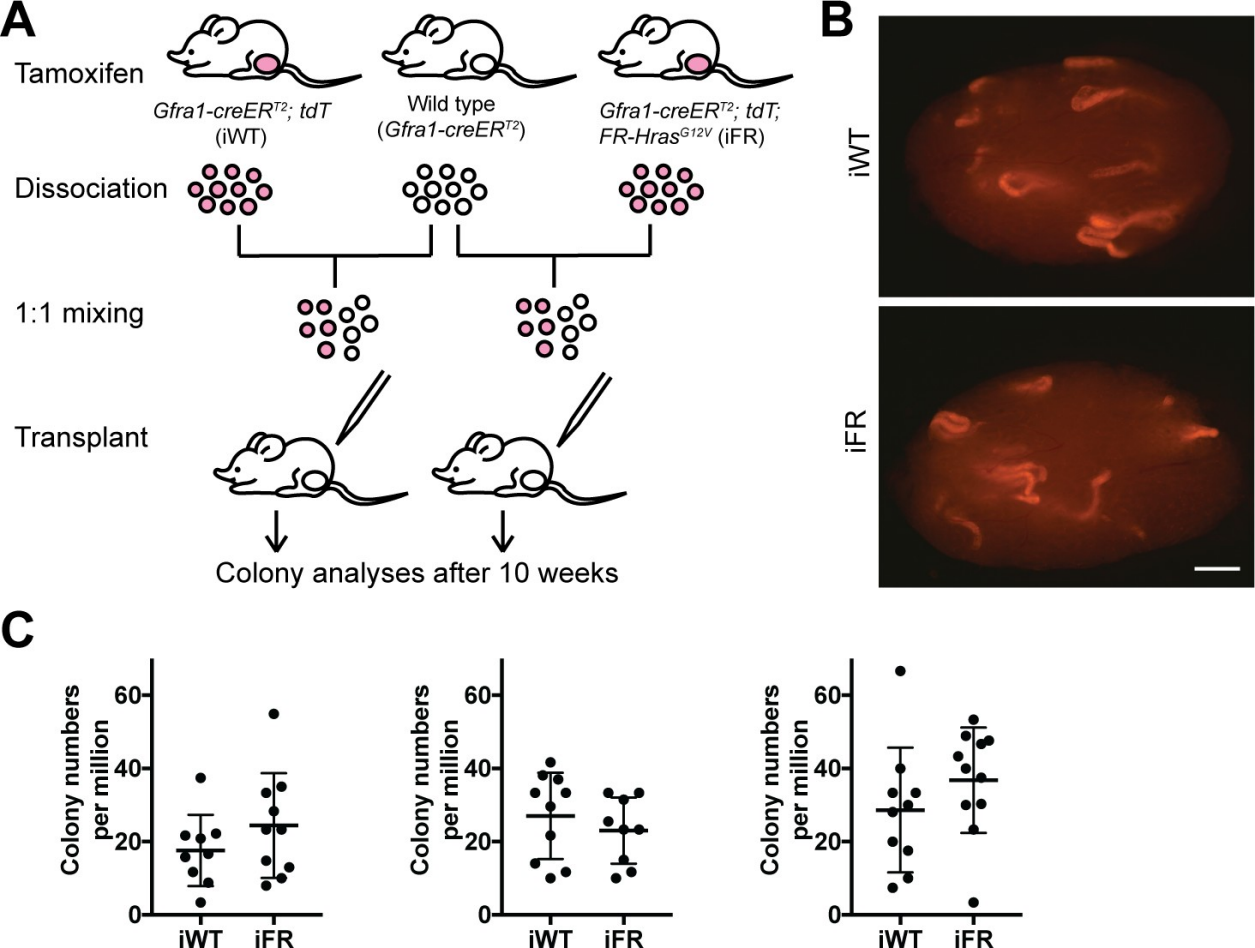
Comparable stem cell capacity in wild-type and *Hras*^G12V^-positive undifferentiated spermatogonia revealed by competitive transplant assay. A. Experimental strategy for competitive transplantation. WT and FR littermates received two 4-day courses of tamoxifen. Two weeks after treatment, testicular cell suspensions from individual iWT, iFR, and non-labeled WT mice were prepared, mixed, and transplanted into sterile recipients. Colony numbers were counted after 10 weeks. B. Representative whole mount stereomicrographs from iWT and iFR transplants after 10 weeks. Scale bar: 1 mm. C. Colony numbers from three independent competitive transplant assays. Colony numbers were similar between iWT and iFR. Each assay used one pair of littermates. T-test (two-tailed, equal variance) was done to compare iWT and iFR (For each experiment, P = 0.25, 0.42, and 0.25, respectively).

To test stem cell capacity of A_undiff_ containing the *Hras*^*G12V*^ mutation, competitive SSC transplant assays developed by Kanatsu-Shinohara et al. (2010) were carried out [30]. In general, transplantation assays serve to measure cell autonomous capacity for self-renewal, survival, and differentiation. By mixing neutral, unlabeled competitor cells with either labeled experimental or labeled control donor cells, respectively, this method is designed to force the donor cells in question to compete against unlabeled competitors and allow direct functional comparisons between the two different donor types. Following two courses of tamoxifen treatment in vivo, the FR locus recombined at high efficiency, concurrent with the *Rosa26-LoxStopLox tdTomato* locus, indicating that most of the A_undiff_ were positive for *Hras*^G12V^ and tdTomato (see next paragraph below). From the previous result above (see Fig 4B), this recombination ratio did not change over an extended period, suggesting that the number of initially induced *Hras*^*G12V*^-positive A_undiff_ remained stable by balancing self-renewal and differentiation for the rest of the time course. Therefore, the timing of testicular cell collection after tamoxifen (i.e., length of chase) should not affect the size of A_undiff_ population. Based on these findings, we chose to perform the transplantation assay 2 weeks after the start of tamoxifen administration. Dissociated testicular cells from these tamoxifen-administered WT (iWT) and FR (iFR) were mixed with non-labeled wild-type competitor testicular cells from *Gfra1-creER*^*T2*^ mice at a ratio of 1:1 and microinjected into busulfan-treated recipient testes (Fig 5A). After 10 weeks, tdTomato-positive colonies were counted (Fig 5B). Colony numbers were comparable between iWT and iFR, suggesting that the short-term cell-intrinsic capacity of stem cells of *Hras*^G12V^-positive A_undiff_ is not greater than that of wild-type cells (Fig 5C).

### Molecular signatures of *Hras*^*G12V*^-positive undifferentiated spermatogonia

**Fig 6:**
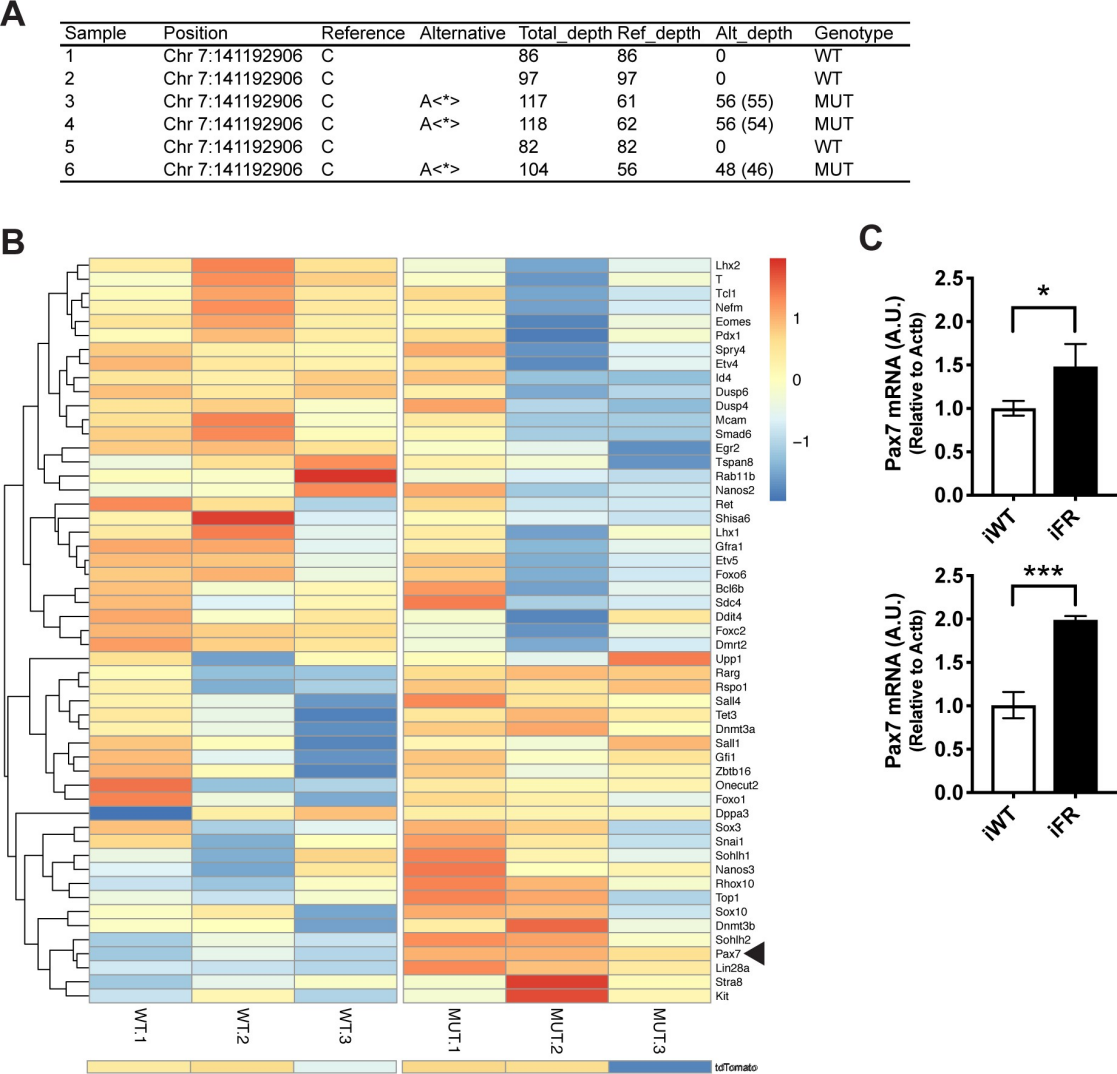
Transcriptome analysis of A_undiff_ from iWT and iFR mice. A. Mutation status by variant calling. In samples 3, 4, and 6 (iFR), almost 50% of the allele (Alt/Total) were *Hras*^G12V^-positive, suggesting most of the A_undiff_ were *Hras*^G12V^+. The filtered values according to quality score are shown in parentheses. Total depth: the total number of reads which map to that position. Ref_depth or Alt_depth: the total number of reads which support the reference or alternative allele. B. Heatmap showing expression of indicated genes from RNA-seq analysis of A_undiff_ from iWT and iFR (MUT) (n = 3 mice per group). The mean was subtracted from each value and divided by the standard deviation (SD). Only *Pax7* (arrow head) was differentially expressed. The first 28 genes are stem cell markers and the rest 25 are differentiation markers, both of which clustered together. tdTomato expression was added on the bottom as a reference. C. Verification of *Pax7* transcript levels (mean±s.d. of technical triplicates) in iWT and iFR by RT-qPCR. Two pairs of animals (top & bottom) were analyzed. In both pairs, *Pax7* was up-regulated in iFR (*P<0.05 and ***P<0.001).

Activating mutations in oncogenes such as RAS family members are thought to produce relatively robust changes in cell phenotype and gene expression, particularly in tumor cells [31, 32]. To understand the molecular features of *Hras*^G12V^+ A_undiff_ in vivo, we isolated tdTomato+ and MCAM bright A_undiff_ (Fig 1A) from WT and FR mice treated with high-dose tamoxifen (i.e., two courses) for RNA-Seq three months after induction. In order to confirm that the *Hras*^G12V^-positive A_undiff_ fraction was sufficiently high in the mutant (MUT) group, a variant calling analysis was performed at position chr7:141192906, where the *Hras*^*G12V*^ (c.35G>T) mutation substitutes an adenine for a cytosine. This revealed that the mutation fraction was 46–49% in the MUT group, indicating that >92% of the isolated A_undiff_ had undergone recombination and were positive for *Hras*^*G12V*^ (Fig 6A). Differential gene expression analysis detected only minimal differences between the two groups, suggesting that A_undiff_ from WT and MUT are highly similar at the transcriptional level (S2 Table). Among the few differentially expressed genes, *Pax7* was found to be upregulated in MUT. This result was confirmed by RT-qPCR (Fig 6C). However, the remaining seven differentially expressed genes were either functionally elusive or apparently irrelevant in A_undiff_. To further capture any subtle differences in phenotype between WT and *Hras*^*G12V*^+ A_undiff_, we manually curated a gene list comprising 28 stem cell marker genes and 25 differentiation marker genes based on recent studies that employed single cell RNA sequencing of mouse A_undiff_ [33, 34] and created a heat map for the differential expression (log_2_ CPM) for each sample (Fig 6B). Apart from *Pax7*, these 52 genes, were not differentially expressed; yet, the stem cell marker genes trended toward slight down-regulation and the differentiation markers exhibited slight up-regulation in the MUT group (Fig 6B). Despite these trends, we concluded that *Hras*^G12V^ does not confer large transcription alterations in mutants.

### Stable phenotype of cultured *Hras*^*G12V*^ SSCs

**Fig 7:**
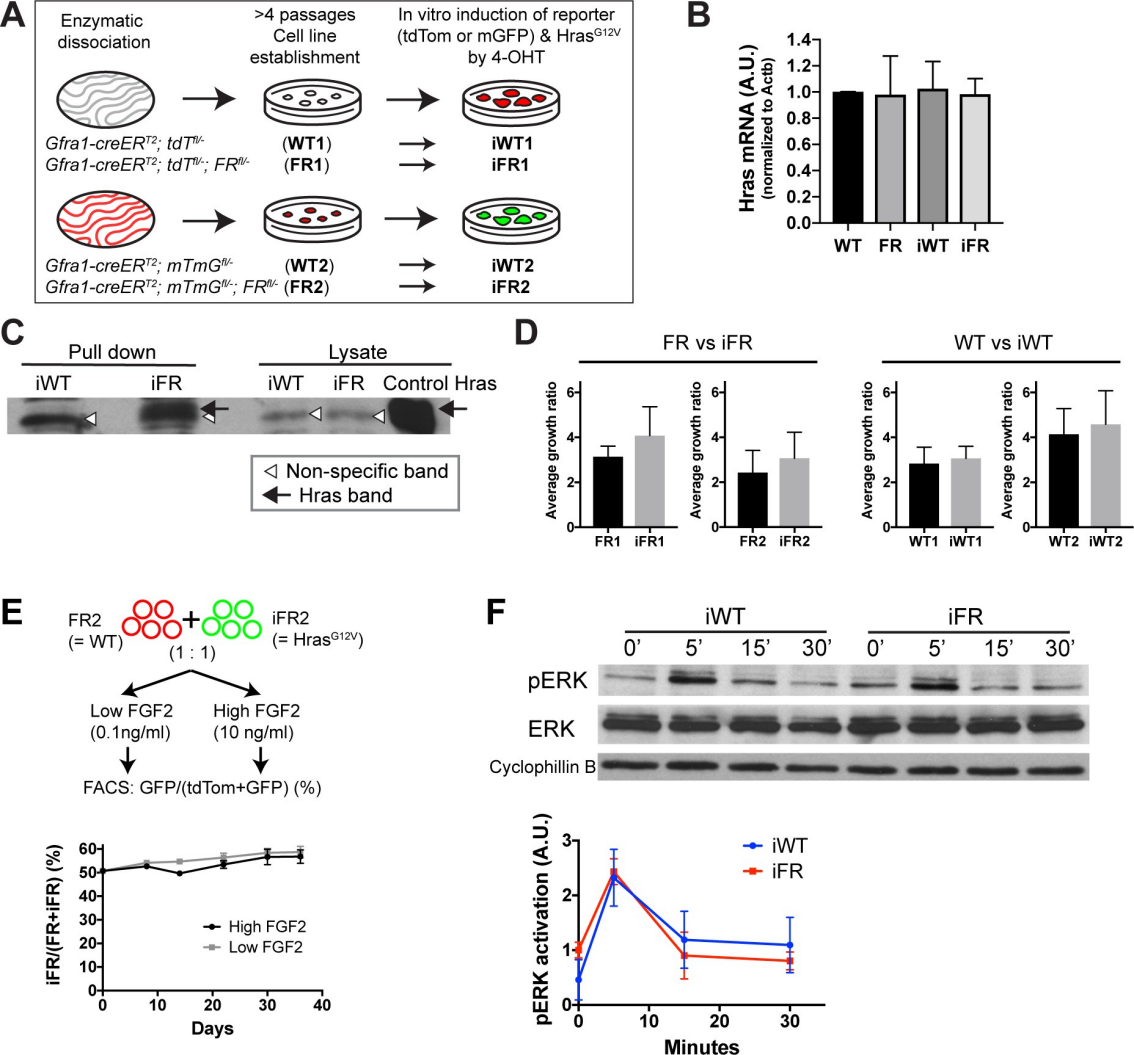
Effect of *Hras*^*G12V*^ on function of cultured SSCs. A. Derivation of cell lines and in vitro induction of *Hras*^G12V^. Two different adult cell line pairs (WT & FR) each from the different reporter systems were established. Dissociated testicular cells were plated and colonies were picked, enriched, and serially passaged on feeders. B. *Hras* transcript levels (RT-qPCR) in different cell lines. Primers were designed to recognize a common region shared by WT and *Hras*^G12V^. N = 3 experiments. C. Western blot for HRAS in SSCs from iWT and iFR. Activated HRAS proteins were pulled down with RAF1 RAS-binding domain and probed by an anti-HRAS antibody (left). In iFR, activated HRAS was observed (black arrow). Open triangle denotes non-specific band. Total lysates (right) from iWT, iFR, and control *Hras* were probed concurrently. Lysate from *Hras*-overexpressing 293T cells served as a positive control. D. Growth ratio of different SSC cell lines. Shown are average weekly growth ratios (4 passages over a month). In all cases, the growth ratios of induced lines were not different from their parental cell lines. E. In vitro competition assay. FR2 (tdTomato+) and iFR2 (GFP+) cells were mixed at 1:1 ratio, allocated to low (0.1 ng/ml) and high (10 ng/ml) FGF2 media, and passaged 5 times. At each passage, GFP+ cell ratios were measured by FACS. F. Phosphorylation of ERK after FGF2 stimulation. Top: Representative western blot (top) from iWT and iFR for pERK, ERK, and loading control, cyclophilin B. Bottom: Quantification based on average densitometric values of western blots from three different sets of clones (bottom). At 0’, iFR exhibited more activation of pErk than iWT (t-test; P = 0.08; n = 3), whereas no significant difference was seen at 5’, 15’ and 20’ between iWT and iFR.

Cultured SSCs serve not only as a complementary model for a complex in vivo system but also enable facile manipulation of extrinsic stimuli to enhance cell phenotypes. Thus, to elaborate on the functional effects of Hras^G12V^ in vitro, we derived SSC cell lines from different adult mouse lines (Fig 7A). Upon 4-OHT addition in vitro, WT cells activated tdTomato (iWT1) or mGFP (iWT2) and FR cells initiated expression of *Hras*^G12V^, in addition to tdTomato (iFR1) or mGFP (iFR2) (Fig 7A). First, the presence of *Hras* transcripts in WT, iWT, FR and iFR lines was measured by designing RT-qPCR primers that recognized a common sequence between the wild-type and *Hras*^*G12V*^ alleles. Levels of *Hras* transcripts were similar in these cell lines, validating that the *SV40* polyadenylation signal in FR does not affect the *Hras* transcript (Fig 7B). Since HRAS^G12V^ protein is locked in a constitutively active conformation, we examined the amount of the functionally active form of HRAS in iFR SSC lines using a pull-down assay, in which only the active form of HRAS is recognized by the RAS-binding domain of RAF1. Active HRAS was pulled down only in the iFR but not in the iWT, demonstrating the presence of functional HRAS^G12V^ protein in this model (Fig 7C).

We next studied changes in the growth trajectory of *Hras*^*G12V*^ SSCs. In normal culture conditions, iFR did not grow faster than FR (Fig 7D). Several lines of evidence show that a fitness advantage may be more pronounced upon environmental challenge, such as growth factor deprivation [13, 35]. We therefore examined whether *Hras*^*G12V*^ SSCs outcompete wild-type SSCs when cultured at an FGF2 concentration 100-fold lower than is used in normal SSC growth media. For the following experiments, FR2 and iFR2 cell lines were employed (Fig 7A & 7E). We mixed the non-induced parental wild-type line FR2 (mtdTomato+) with iFR (mGFP+) at a 1:1 ratio and cultured them on feeder cells in FGF2 (0.01 or 10 ng/ml) for up to five passages. iFR did not replace its wild-type parental line over time (Fig 7E). This result suggests that a low FGF2 environment does not confer a competitive advantage to *Hras*^*G12V*^ SSCs.

*Hras* is an important mediator molecule of FGF-RAS-MAPK signaling [23], and FGF2 has been shown to control stem cell self-renewal and possibly differentiation in SSCs [36, 37]. In various RASopathy models, RAS-MAPK signaling pathways are highly dysregulated [22, 38, 39]. However, it is unclear whether this holds true for SSCs harboring endogenous gain-of-function mutations seen in RASopathies. To understand how MAPK signaling is altered in *Hras*^*G12V*^ SSCs in response to FGF2, iWT and iFR SSC clones were stimulated with FGF2 and probed for pERK and total ERK. pERK was similarly phosphorylated in iWT and iFR in response to FGF2 (Fig 7F). However, basal level ERK phosphorylation prior to stimulation appeared to be slightly higher in iFR as compared to iWT. Lack of enhanced ERK activity in iFR following stimulation was unexpected, considering the presence of active HRAS protein in iFR cell lines. This result suggests that active HRAS at a physiological gene dosage does not augment the signal response to FGF2 in SSCs.

## Discussion

The competitive interactions of wild-type and neighboring mutant germline stem cells in the adult human testis are thought to produce uneven transmission of pathogenic alleles to children, but a dearth of model systems has hindered progress toward understanding the details of selection. In this study, we created a stem cell competition system in the mouse germline, in which undifferentiated spermatogonia with endogenous expression of an oncogenic mutation, *Hras*^*G12V*^, compete with their wild-type counterparts in the testis. We found that the *Hras*^*G12V*^ mutation was transmitted from the paternal germline stem cells to offspring, whereas the mutated germ cells did not exhibit a significant expansion over a year of fate analysis.

For in vivo genetic manipulation of the A_undiff_, which contain the stem cell pool, only a handful cre driver lines have been described, including Vasa-cre and Stra8-cre. These cre alleles are not only non-specific with respect to germ cell subpopulations but also are active during early gonadal development, making it difficult to study adult mouse SSCs. For these reasons, cultured SSCs have been employed for loss- or gain-of-function studies using knock-down/out or ectopic expression of genes [13, 23]. Hara et al. (2014) first leveraged *Gfra1-creER*^*T2*^ for lineage tracing of adult A_undiff_; by measuring long-term self-renewal of the population, they demonstrated that genetically marked A_undiff_ are functional stem cells that persist at homeostatic levels [16]. In our mouse model, therefore, we adapted *Gfra1-creER*^*T2*^ to induce both a mutation and a label in the A_undiff_ and achieved robust recombination at the target loci in A_undiff_. Conveniently, the size of the recombined fraction was adjustable from ~8 to almost 100% by dose of tamoxifen. This tunability makes *Gfra1-creER*^*T2*^ an appropriate cre-driver to study germline mosaicism, in which only a small subset of stem cells is genetically manipulated or marked and their fate is traceable thereafter. On the other hand, with a high dose of tamoxifen, *Gfra1-creER*^*T2*^ is suitable to obtain a highly enriched population of genetically manipulated germline cells in vivo. Indeed, by combining *Gfra1-creER*^*T2*^ with a reporter and anti-MCAM staining, we were able to enrich an A_undiff_ sample with 96% *Hras*^*G12V*^ positivity, as measured at the RNA level. Although *Gfra1-creER*^*T2*^ seems to be best available inducible driver to genetically manipulate adult A_undiff_, it is notable that *Gfra1* is broadly expressed in the A_undiff_ and that the *Gfra1*-expressing population comprises a heterogeneous subset of A_undiff_. It remains controversial whether *Gfra1*-negative stem cells exist in the A_undiff_.

Complex engineered alleles are required to model precisely the genetics of human germline disorders. In the *FR-Hras*^*G12Vfl/-*^ system, we sought to induce *Hras*^*G12V*^ without affecting total gene dosage throughout the recombination process; accordingly, similar levels of *Hras* transcript were observed before and after cre induction. Thus, such an approach most closely recapitulates the occurrence of human de novo mutations in male germline stem cells and the resultant germline mosaicism. Also, since this genetic model entails two tandem *Hras* genes, both comprising endogenous exons and introns, the final transcription product is also an endogenously spliced variant, in case any alternatively spliced SSC-specific variant exists. In our model, we found that recombination at the *Rosa26* reporter locus happened more efficiently than that of the *FR-Hras*^*G12V*^ locus. This could be explained by accessibility of the locus to cre recombinase; the *Rosa26* locus is known for its constitutively active promoter region [40], whereas the activity of the *Hras* locus may be tightly regulated by transcription factors and/or chromatin remodeling.

Classical PAE disorders, such as Noonan syndrome, Costello syndrome, and other RASopathies, are caused by de novo mutations that are found only in affected children but not in the somatic DNA of either parent. Although most such mutations are derived from the paternal germline (as reviewed in [6]), the process of transmission from paternal germline stem cells to the offspring has not been widely explored. A major strength of our strategy is that it allows one to interrogate the transmission of the mutated gene induced in male germline stem cells to subsequent generations. Here, we observed that the fate of *Hras*^G12V^ stem cells resulted in *Hras*^G12V^ offspring with neonatal mortality, consistent with Costello syndrome. This indicates that A_undiff_ carrying *Hras*^*G12V*^ can differentiate into functional sperm, and the resultant Costello embryos are capable of developing to the neonatal stage. Similarly, our germline mosaic model could be applied to a variety of other de novo disorders with PAEs. Owing to technological advancements in genome sequencing, many de novo mutations that originate in the paternal germline have been implicated as drivers of neurodevelopmental disorders (e.g., autism). Yet, adult germline mosaicism as a major source of human disease is still controversial, and its natural course has not been addressed experimentally. By inducing specific autism-driving mutations in A_undiff_ and tracking their fate, our mosaic model could be useful going forward to uncover the causal relationship between paternal age and neurodevelopmental disorders.

A human sperm analysis revealed that healthy males carried different *Hras* mutations at codon 12 [9]. Although *G12S* was the most prevalent mutation, *G12V* was also substantially elevated in the sperm and the level was weakly correlated with donor age. Thus, we sought to investigate how *G12V*-laden germline cells expand in our experimental model. To perform stringent lineage tracing of *Hras*^G12V^-positive cells, we employed multiple independent quantification methods and their outcomes were well correlated: cDNA sequencing from labeled germ cells, sperm gDNA analysis, and sperm counting. Over a year of lineage tracing, we did not observe a significant increase in the *Hras*^G12V^ cell and sperm populations. In the serial sperm analysis, a minimal increase (~9%) was observed over the 8–9 month period, yet we did not observe an increase in the proportion of *Hras*^G12V^ offspring from mutant sires in successive litters. Despite the fact that the *Hras*^G12V^ is the most potent gain-of-function mutation among various HRAS proteins at codon 12, these findings indicate that the heterozygous *Hras*^*G12V*^ alone is not sufficient to drive SSC competition in the mouse testis.

There are several possible explanations for the absence of competition in our model. First, there could be fail-safe mechanisms to suppress aberrant cell signaling driven by hyperactive RAS, providing a robust system that protects stem cells from harmful consequences. Second, HRAS may not have a major role in FGF-RAS-MAPK signaling in the A_undiff_, which would be unexpected given previously published data [23]. Other members of the RAS superfamily may be critical for relaying FGF signals in the SSCs. Third, the *Hras*^*G12V*^ mutation may be detrimental, such that that cell turnover could be faster than usual. Fourth, induction experiments performed at extremely low starting (i.e., baseline) mutation levels (e.g., <1% recombination) might reveal competitive interactions that could have been obscured in our experiments, due to as yet uncharacterized paracrine effects. Fifth, key differences could exist in the microenvironment or cellular population structure between mice and humans. Sixth, the observation period in this study (~one year) could be too short to capture long-term cell competition because of the much shorter lifespan of mice than of humans, which entails decades of continuous stem cell survival and self-renewal in the testis. Regarding these last two points, age-related changes in the stem cell niche could be necessary for *Hras*^*G12V*^-mediated stem cell competition to become apparent. Finally, additional, yet unidentified genetic lesions could be required to confer enhanced competitiveness.

An absence of cell competition was also observed in vitro. In cultured SSCs with *Hras*^*G12V*^, although a functionally active HRAS was detected, ERK activation following FGF2 stimulation was not enhanced. Chen et al. (2009) made a similar observation, using mouse embryonic fibroblasts (MEFs) with heterozygous *Hras*^*G12V*^ [22]. Only in later passaged *Hras*^*G12V*^ MEFs was more pERK detected than in controls. This suggests that active HRAS from one copy of *Hras*^*G12V*^ is not adequate to activate ERK in either cultured SSCs or MEFs, and more time may be required to gain additional gene mutational hits. Furthermore, a reduced FGF2 environment did not favor selection of the *Hras*^*G12V*^ cell population, indicating that HRAS may not be a major GTPase mediating FGF2 signaling in SSCs. On the other hand, when *Hras*^G12V^ is overexpressed in cultured SSCs, increased proliferation and oncogenic transformation were observed [23], again suggesting that the *Hras*^*G12V*^ effect is dependent on gene dosage.

The transcriptional profile between wild-type and *Hras*^G12V^ A_undiff_ did not reveal a significant difference in gene expression. Interestingly, among a few differentially expressed genes, *Pax7* was up-regulated in iFR cells. *Pax7* is a transcription factor identified as a conserved marker for a particularly rare subset of A_undiff_ in mammalian testes [33, 41]. Although its function in self-renewal of A_undiff_ is not well characterized, *Pax7* may be one of the downstream genes upregulated by MAPK signaling via HRAS in a subset of the A_undiff_ population. Overall, the absence of large transcriptional changes in *Hras*^G12V^ A_undiff_ could account for the fact that there was no obvious competitiveness or higher stem cell capacity in *Hras*^*G12V*^-positive A_undiff_ in the transplant assays.

In conclusion, these results revealed a stable and tolerant system to prevent normal germline stem cells from being replaced by mutated cells. This unanticipated resistance to hyper-active *Hras* suggests inherent mechanisms within germline stem cells to suppress harmful mutations that would otherwise be propagated to offspring. In contrast, we anticipate that future studies will likely uncover factors that overcome such protective mechanisms, leading to aberrant clonal expansion. Given the increasing number of disorders (e.g., autism) linked to germline mosaicism and PAEs, the inducible adult mosaic model will be invaluable to understand the earliest origins of such pathogenic gene variants.

## Materials and methods

### Ethics statement

This study was approved by the Weill Cornell Medical College IACUC (#2010–0028). Either isoflurane or Ketamine/Xylazine was used for anesthesia in combination with buprenorphine and meloxicam for analgesia. For euthanasia, mice were exposed to CO2 followed by cervical dislocation.

### Animals

*Gfra1-creER*^*T2*^ mice were a gift from Dr. Sanjay Jain [16, 42]. Reporter mice, *tdTomato* (#007914) [43] and mT/mG (#007676) [29], were obtained from the Jackson Laboratory. The *FR-Hras*^*G12Vfl/fl*^ mice were previously generated by Dr. James Fagin [22]. The controls were wild-type littermates that do not have the *FR-Hras*^*G12Vfl*^ allele but contain *Gfra1-creER*^*T2*^ to induce tdTomato expression. These mice were maintained on a mixed genetic background of C57BL/6J (>50%), 129/Sv, and Swiss Black mice. All the experimental protocols were approved by the Weill Cornell Medicine Institutional Animal Care and Use Committee.

### Lineage tracing

At 6–8 weeks of age, 100 mg/kg of tamoxifen (Sigma) dissolved in corn oil (Sigma) was administered intra-peritoneally for 4 days (one standard course), unless otherwise specified. At each time point, animals were euthanized and the testes and caudal epididymal sperm were harvested for downstream experiments.

### Immunofluorescence

Detunicated testes were fixed with 4% paraformaldehyde in phosphate buffered saline overnight at 4°C, immersed in 30% sucrose, and embedded in OCT compound. After cryosectioning the samples at 10 μm, DAPI was applied. For whole-mount staining, after overnight fixation, the seminiferous tubules were untangled, washed in PBS, and blocked with 3% BSA/PBS with 0.1% Tween for an hour. After incubation with anti-Gfra1 antibody (1:200, BD) overnight, an anti-rabbit biotinylated secondary antibody, followed by Alexa647-conjugated streptavidin was used for detection. DAPI was used for nuclear staining. Images were captured with a Zeiss LSM 800 confocal microscope.

### Serial epididymal sperm sampling and sperm gDNA extraction

Microsurgical epididymal sperm aspiration was performed as previously described [28]. Under deep anesthesia, through a small skin incision on the scrotum, the cauda epididymis was punctured by a syringe, and its contents were aspirated (S2 Fig). The procedure was performed on ipsilateral testis at 4 months and again at 12 months after tamoxifen administration. gDNA extraction was performed using AllPrep DNA/RNA Mini Kit (Qiagen) with a modified protocol [44].

### FACS

A whole testis dissociate was prepared using a two-step enzymatic digestion [45]. For MCAM staining, testes from *Gfra1-creER*^*T2*^; *tdTomato* mice (n = 3) were dissociated >3 months after tamoxifen administration. The single-cell suspensions from two testes were incubated with Alexa Fluor 647 anti-MCAM antibody (ME-9F1, BioLegend) at a concentration of 6 g/ml for 45 minutes at 4°C. After exclusion of doublets and DAPI-positive cells, Alexa Fluor 647 and tdTomato double-positive cells were gated and collected using a BD Aria flow cytometer.

### RT-qPCR

Total RNA was extracted from sorted testicular cells or feeder-free cultured SSCs using Arcturus PicoPure Kit (Applied Biosystems) or RNeasy Plus micro kit (Qiagen), respectively, with an on-column DNA digestion protocol (Qiagen). Reverse transcription was performed using qScript (Quanta Biosciences) followed by a real-time PCR using Sybr Select Master Mix (Applied Biosystems) with a LightCycler 480II (Roche). Each technical triplicate was normalized to *Actb* and relative expression levels to control conditions were calculated using 2^-ΔΔCt^ method.

### Quantification of the *Hras*^G12V^-positive cell fraction using Sanger sequencing traces

FACS-collected tdTomato+ cells were lysed in RLTplus buffer (Qiagen RNA mini kit) with 2% β-mercaptoethanol, and RNA was purified according to the manufacturer’s instructions. The RNA was reverse-transcribed to cDNA using qScript cDNA SuperMix (Quanta Biosciences). A targeted region flanking *Hras*^*G12V*^ was amplified by PCR and Sanger-sequenced. To quantify the mutated nucleotide (cytosine→adenine) from Sanger sequencing traces, a model curve was proposed and confirmed by amplifying cDNA samples of known concentrations from WT (*Hras*^WT/WT or FR/WT^) and Costello offspring (*Hras*^*G12V/WT*^) RNA (x-axis: 0, 25, 50, 75, and 100% of Costello RNA) (Fig 2A). By using 4 pairs of different offspring samples, it was confirmed that the relationship between the *Hras*^G12V^-positive cell fraction (x%) and ratio of adenine/cytosine (A/C) peak heights (y) fitted the model curve y = 1.371*x / (200-x). All the calculations for *Hras*^G12V^-positive cell fraction were done using this equation. ImageJ was used to measure A/C ratio via peak height. All the primers used in the study are listed in S3 Table.

### Quantification of the *Hras*^G12V^+ cell fraction by gDNA qPCR

To obtain a fractional abundance of the *Hras*^*G12V*^ allele, qPCR primers were designed, detecting the SV40 PA region that is exclusively present in the *FR-Hras*^*G12Vfl*^ locus and lost after recombination (Fig 2B). By running qPCR with mixed gDNA from a *FR-Hras*^*G12V*^ heterozygote (without recombination) and wild-type sperm collected from cauda epididymides at different known ratios (x: 0, 25, 50, 75, 100% of *FR*^*fl/-*^ allele), we confirmed a linear relationship between the *FR-Hras*^*G12Vfl*^ recombined fraction (x%) and the SV40 PA recombined fraction obtained by qPCR (y%) (Fig 2B left). Since the loss of SV40 PA or FR means a gain of *Hras*^*G12V*^, y = -z+100 was obtained, where z is the *Hras*^G12V^+ cell fraction (%) (Fig 2B, right). Per reaction, 60ng of sperm gDNA in 4 ul was used. For normalization of the quantity of gDNA, *Ngn3* gene primer sets were used. Light Cycle480 Software was used for analysis. To quantify a recombined fraction of a reference gene, *mTmG*, we employed the same strategy as the quantification for *Hras*^G12V^+ cell fraction. Primer sets were designed to detect a region of the mTomato locus that is deleted after recombination (Fig 2B).

### Sperm counting

Sperm obtained by microsurgical epididymal sperm aspiration was mounted on slides and imaged using a BX50 fluorescence microscope (Olympus) with a Spot Pursuit CCD camera (Diagnostic Instruments Inc). Total of 100 to 300 sperm (> 3 fields) were counted.

### Competitive transplant assays

To obtain the highest labeling after the maximum dose of tamoxifen, testis cell suspensions from *Gfra1-creER*^*T2*^; *tdTom* mice and *Gfra1-creER*^*T2*^; *tdTom; FR-Hras*^*G12V*^ mice (littermate of the former) were mixed with that of non-labeled wild-type (*Gfra1-creER*^*T2*^) at a 1:1 ratio, respectively, and transplanted into adult busulfan-conditioned C57Bl6 recipient testes (n = 10 or 11 mice per experiment). A total of 1.2 x 10^6^ (first two experiments) or 0.6 x 10^6^ (the 3^rd^ experiment) mixed cells were injected per testis. Site of injection (left vs. right side) was alternated per genotype. Three independent transplantations were performed (3 donor mice per group). After 10 weeks, colony numbers were quantified using stereomicroscopy.

### RNA-seq

WT and FR mice (n = 3/ group) were treated with tamoxifen for four days for twice over two weeks. The testes were dissociated (see Fig 1A) and RNA from tdTomato+ MCAM-bright A_undiff_ was isolated via Arcturus PicoPure kit with DNase I treatment. Mean RNA Integrity Number (RIN) was 9.47 (SD 0.31). Libraries were constructed using TruSeq Stranded mRNA. Sequencing was performed on an Illumina HiSeq 2500 (v4 chemistry) with a 50 bp paired-end protocol. Variant calling and filtering were carried out using *Bam-Readcount* and *SAMtools mpileup* at chr7:141192906, where samples with heterozygous *Hras*^*G12V*^ (c.35G>T) mutation should have evidence of a C/A genotype while wild-type samples have only a C genotype. Differential expression was assessed using DESeq2 with a false discovery rate (FDR) of 0.1.

### Derivation and culture of adult SSC lines and in vitro 4-OHT induction

SSC lines were derived from two pairs of littermate adult mice (see Fig 7A for specific cell lines) and maintained on mitotically-inactivated JK1 feeders [46]. SSC growth media was StemPro-34 with additional supplements as described previously [45]. Treatment with 6 μM of 4-OHT for 4 days was repeated for a total of 3–4 times over 3–4 weeks until the *Hras*^G12V^ population was confirmed to be more than 70–80% by the sequencing method (see Fig 2A–2C). Between tamoxifen treatments, tdTomato-positive cells were enriched once by FACS to enhance the recombination efficiency for *Hras*^*G12V*^. All the experiments were performed using cells with passage number 9 to 22. The stem cell activity was confirmed by transplantation assays.

### Active HRAS pull-down

An HRAS activation assay was performed using a GST-fusion protein of the RAS-binding domain (RBD) of RAF1, as instructed by the manufacturer (Pierce Biotechnology). Briefly, SSCs maintained in growth media were washed with cold TBS and lysed. Active RAS was pulled down with GST-RAF1-RBD along with glutathione agarose resin, followed by Western blot detection with an anti-Hras antibody (sc-520, Santa Cruz Biotechnology).

### Immunoblotting

After feeder subtraction, SSCs were washed once with ice-cold TBS and lysed in lysis buffer containing PMSF (Sigma), protease inhibitor (Sigma), and phosphatase inhibitor cocktails (Sigma). Protein concentration was quantified by the BCA method. Immunoblotting was performed according to standard procedures. Denatured samples were subjected to 12% SDS/PAGE gel and transferred to PVDF membrane. The following antibodies were used: pERK (9101, Cell signaling), ERK (9102, Cell Signaling), CyclophilinB (Invitrogen), and Hras (sc-520). To obtain mouse HRAS protein control for western blotting, a fragment (606 bp) of *Hras* (NM_008284.2) was synthesized by Integrated DNA Technologies, inserted into BamHI/EcoRI sites of pCIG [47], and overexpressed in HEK293T cells.

### In vitro competition assay

*Gfra1-creER*^*T2*^*; mTmG*^*fl/-*^*; FR*^*fl/-*^ (FR2: tdTomato+ wild-type cells) and its induced derivative iFR2 (GFP+ *Hras*^G12V^) cells were mixed at 1:1 ratio, cultured in low (0.1 ng/ml) and high (10 ng/ml) FGF2 media, and passaged 5 times. At each passage, GFP+ cell ratios were measured by FACS (BD Accuri).

### Agonist-invoked signaling assay

Feeder-free SSCs were starved for 18 hours and then either left unstimulated (0’) or stimulated with FGF2 (10 ng/ml), and harvested 5, 15, and 30 minutes after the stimulation. Cell lysates were analyzed by immunoblotting.

### Statistics

Results are presented as mean ± SD. At least three biological replicates and three technical replicates were performed for each experiment unless otherwise indicated in the text. GraphPad Prism was used for statistical analyses and generating graphs.

## Supporting information

S1 Fig Generation of a model equation for quantification of the *Hras*^G12V^+ cell fraction using RNA samples. Five different conditions of recombination status (0–100% of *Hras*^*G12V*^) are shown. In an ideal situation, the A/C ratio reflects the ratio of mutant/WT mRNA. However, after reverse transcription, PCR, and Sanger sequencing, the difference in amplification efficiency between A and C is pronounced, which is expressed as constant *k*.
(PDF)

S2 Fig Microsurgical epididymal sperm aspiration. Schematic illustrating how sperm is aspirated from the cauda epididymis. A small skin incision is made over the caudal pole of the testis and the cauda epididymis is identified. The fascia is kept intact throughout the procedure. The cauda epididymis is retracted and gently squeezed by the curved serrated forceps. Using an insulin syringe (29-30G) loaded with a small amount of PBS, the epididymis is punctured through the fascia and the sperm are aspirated and transferred into a plate. This is repeated several times to obtain a sufficient amount of sperm. If the cauda epididymis is difficult to locate, the fascia is opened and the cauda epididymis can be punctured directly. The skin (and fascia, as well if it is opened) is sutured after the procedure.
(PDF)

S1 Table Analysis of offspring obtained at early vs. late periods from tamoxifen-induced HrasG12V sires. A total of 217 offspring from three iFR sires were analyzed for their genotypes. To investigate paternal age effect, we tested whether the number of Costello offspring born was different between an early and a late period of breeding. Costello and non-Costello offspring numbers were allocated into two groups: those born before (early) and after (late) day 210 after tamoxifen.
(PDF)

S2 Table Differentially expressed genes. Genes that were differentially expressed in the RNA-seq analysis.
(PDF)

S3 Table Primer list. Genes and corresponding primers used in this study.
(PDF)
