# Supplementary figures and images for: Functional robustness of adult spermatogonial stem cells after induction of hyperactive Hras
Source: PLoS Genet. 2019 May 3;15(5):e1008139. doi: 10.1371/journal.pgen.1008139 (PMC6519842; doi:10.1371/journal.pgen.1008139)

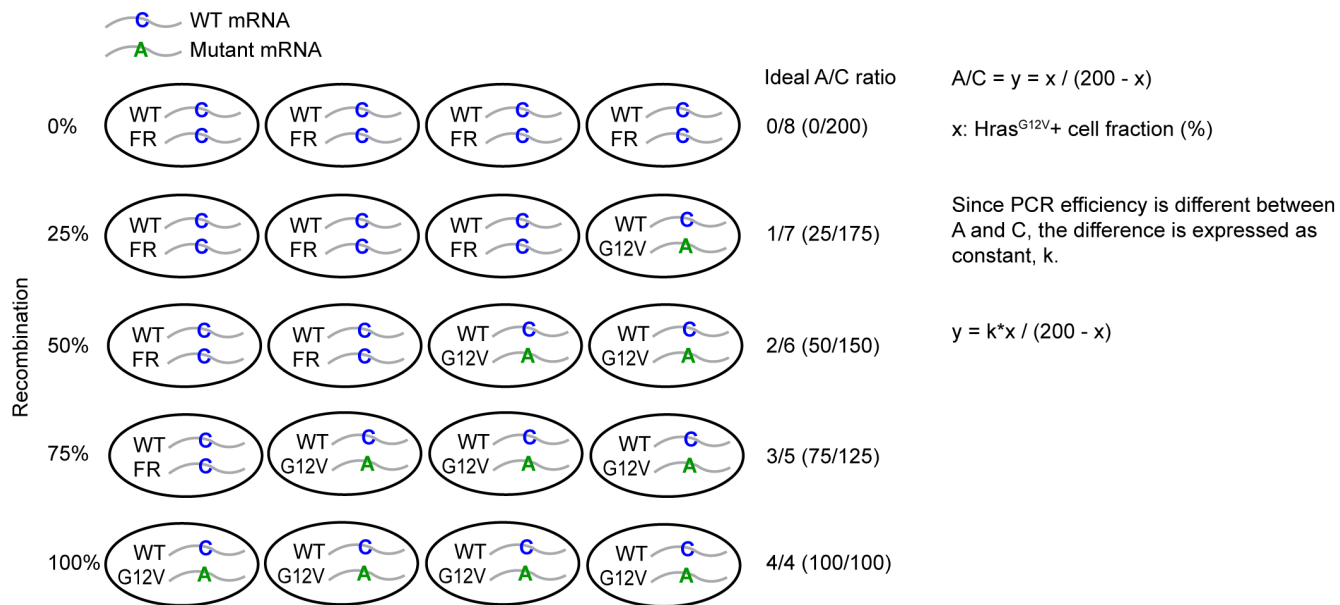

Supplement: S1 Fig — Five different conditions of recombination status (0–100% of HrasG12V) are shown. In an ideal situation, the A/C ratio reflects the ratio of mutant/WT mRNA. However, after reverse transcription, PCR, and Sanger sequencing, the difference in amplification efficiency between A and C is pronounced, which is expressed as constant k. (PDF) [file pgen.1008139.s001.pdf]

Microsurgical epididymal sperm aspiration

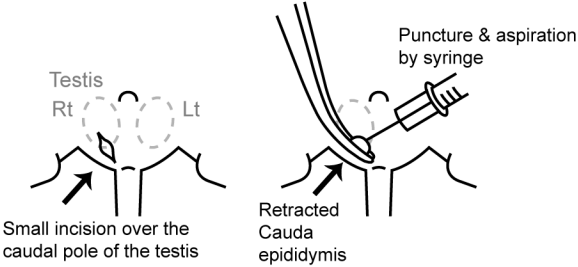

Supplement: S2 Fig — Schematic illustrating how sperm is aspirated from the cauda epididymis. A small skin incision is made over the caudal pole of the testis and the cauda epididymis is identified. The fascia is kept intact throughout the procedure. The cauda epididymis is retracted and gently squeezed by the curved serrated forceps. Using an insulin syringe (29-30G) loaded with a small amount of PBS, the epididymis is punctured through the fascia and the sperm are aspirated and transferred into a plate. This is repeated several times to obtain a sufficient amount of sperm. If the cauda epididymis is difficult to locate, the fascia is opened and the cauda epididymis can be punctured directly. The skin (and fascia, as well if it is opened) is sutured after the procedure. (PDF) [file pgen.1008139.s002.pdf]
