# Supplementary material for: Functional robustness of adult spermatogonial stem cells after induction of hyperactive Hras
Source: PLoS Genet. 2019 May 3;15(5):e1008139. doi: 10.1371/journal.pgen.1008139 (PMC6519842; doi:10.1371/journal.pgen.1008139)
Supplement: S1 Table — A total of 217 offspring from three iFR sires were analyzed for their genotypes. To investigate paternal age effect, we tested whether the number of Costello offspring born was different between an early and a late period of breeding. Costello and non-Costello offspring numbers were allocated into two groups: those born before (early) and after (late) day 210 after tamoxifen. (PDF) [file pgen.1008139.s003.pdf]

S1 Table. Analysis of offspring obtained at early vs. late periods from tamoxifen-induced HrasG12V sires.

Interval	Genotype		
	Costello	Non-Costello	Row totals
≤210 day*	35	58	93
>210 day	48	76	124
Column totals	83	134	217
The chi-square statistic is 0.026. *p*= .871865.			
*The day 210 was chosen because it was a mid-point of the whole period of breeding.			
