# Supplementary material for: Functional robustness of adult spermatogonial stem cells after induction of hyperactive Hras
Source: PLoS Genet. 2019 May 3;15(5):e1008139. doi: 10.1371/journal.pgen.1008139 (PMC6519842; doi:10.1371/journal.pgen.1008139)
Supplement: S2 Table — Genes that were differentially expressed in the RNA-seq analysis. (PDF) [file pgen.1008139.s004.pdf]

S2 Table. Differentially expressed genes.

Gene	baseMean	log2FoldChange	lfcSE	stat	pvalue	padj
Igkv14-111	31.46841245	-8.420579982	1.30855229	-6.4350352	1.2344E-10	2.3395E-06
Ighv3-6	23.46902678	-7.99763176	1.2645373	-6.3245519	2.5397E-10	2.4066E-06
Igkv4-91	32.0291505	-7.477124742	1.23809128	-6.0392354	1.5485E-09	9.7821E-06
Ighv1-55	22.25762518	-7.922019636	1.3716479	-5.775549	7.6703E-09	3.6342E-05
Ighv1-64	11.58507694	-6.9789357	1.38336476	-5.0448992	4.5376E-07	0.00171993
Pax7	183.5334767	0.948479756	0.19466157	4.87245514	1.1022E-06	0.00348148
hexb	924.6607351	-0.604721061	0.13004709	-4.6500159	3.3191E-06	0.00898621
Angptl7	31.28563471	-1.979457698	0.461786	-4.286526	1.8149E-05	0.04299474
