# Supplementary material for: Functional robustness of adult spermatogonial stem cells after induction of hyperactive Hras
Source: PLoS Genet. 2019 May 3;15(5):e1008139. doi: 10.1371/journal.pgen.1008139 (PMC6519842; doi:10.1371/journal.pgen.1008139)
Supplement: S3 Table — Genes and corresponding primers used in this study. (PDF) [file pgen.1008139.s005.pdf]

S3 Table. Primer list.

Name	Purpose	Amplicon size	Forward	Reverse
Hras F1 & R1	cDNA PCR	313	GCCGCTGTAGAAGCTatgAC	TCTGCTCCctgtactgatgg
Hras R2	Sanger sequencing	N/A	N/A	cctgctgtgtctaagatgtcc
Ngn3	gDNA qPCR	155	aga gcg agt tgg cac tca gc	ctc gat ctt tgt aag ttt ggc g
Nanos2	gDNA qPCR	116	agg aga aga gtg agc agg ac	cgt ctt cag ctg gtg tga gg
SV40 polyA of FR-Hras	gDNA qPCR	76	TCTGGCTGGAAGTAGGGTCG	ACTGCATTCTAGTTGTGGTTTGT
mTmG	gDNA qPCR	93	ATGGGAGCAGTGGTGGAATG	TGAGAGTCAGCAGTAGCCTCA
Hras	RT-qPCR	92	GGAAAGAGTGCCCTGACCAT	ACCACCTGTTTCCGGTAGGA
Actin beta	RT-qPCR	132	GAGAAGATCTGGCACCACACC	GGTCTCAAACATGATCTGGGTC
Gapdh	RT-qPCR	120	CTAACATCAAATGGGGTGAGG	CGGAGATGATGACCCTTTTG
Gfra1	RT-qPCR	89	AGAGCCTGCAGGGAAATGAC	GAACGGGACTGCCCGGAATA
Pax7	RT-qPCR	169	GTGCCCTCAGTGAGTTCGAT	CCACATCTGAGCCCTCATCC
